# Epigenetic-Mediated Downregulation of *μ*-Protocadherin in Colorectal Tumours

**DOI:** 10.1155/2015/317093

**Published:** 2015-04-20

**Authors:** Bujko Mateusz, Kober Paulina, Statkiewicz Małgorzata, Mikula Michal, Ligaj Marcin, Zwierzchowski Lech, Ostrowski Jerzy, Siedlecki Janusz Aleksander

**Affiliations:** ^1^Department of Molecular and Translational Oncology, Maria Sklodowska-Curie Memorial Cancer Center and Institute of Oncology, 5 W.K. Roentgena, 02-781 Warsaw, Poland; ^2^Department of Genetics, Maria Sklodowska-Curie Memorial Cancer Center and Institute of Oncology, 5 W.K. Roentgena, 02-781 Warsaw, Poland; ^3^Department of Pathology, Maria Sklodowska-Curie Memorial Cancer Center and Institute of Oncology, 5 W.K. Roentgena, 02-781 Warsaw, Poland; ^4^Department of Molecular Biology, Institute of Genetics and Animal Breeding, 36A Postępu 36A, Jastrzębiec, 05-552 Magdalenka, Poland

## Abstract

Carcinogenesis involves altered cellular interaction and tissue morphology that partly arise from aberrant expression of cadherins. Mucin-like protocadherin is implicated in intercellular adhesion and its expression was found decreased in colorectal cancer (CRC). This study has compared *MUPCDH* (*CDHR5*) expression in three key types of colorectal tissue samples, for normal mucosa, adenoma, and carcinoma. A gradual decrease of mRNA levels and protein expression was observed in progressive stages of colorectal carcinogenesis which are consistent with reports of increasing *MUPCDH* 5′ promoter region DNA methylation. High *MUPCDH* methylation was also observed in HCT116 and SW480 CRC cell lines that revealed low gene expression levels compared to COLO205 and HT29 cell lines which lack DNA methylation at the *MUPCDH* locus. Furthermore, HCT116 and SW480 showed lower levels of RNA polymerase II and histone H3 lysine 4 trimethylation (H3K4me3) as well as higher levels of H3K27 trimethylation at the *MUPCDH* promoter. *MUPCDH* expression was however restored in HCT116 and SW480 cells in the presence of 5-Aza-2′-deoxycytidine (DNA methyltransferase inhibitor). Results indicate that *μ*-protocadherin downregulation occurs during early stages of tumourigenesis and progression into the adenoma-carcinoma sequence. Epigenetic mechanisms are involved in this silencing.

## 1. Introduction

The intestinal epithelium is a complex structure undergoing dynamic change both during development and throughout its lifetime, until becoming shed into the intestinal lumen. Epithelium formation and renewal requires strict coordination of cellular migration and adhesion. Molecules forming cellular adhesion junctions not only are structural in function, but also play an important role in intercellular and intracellular signalling. The loss of control over adhesion and migration invariably leads to tumourigenesis and the acquiring of a tumour aggressive phenotype. Aberrant expression of cell-cell adhesion related proteins, including the well described cadherin switch phenomenon, has been reported in various human tumours [1, 2].

The cadherin superfamily contains transmembrane proteins involved in cell-cell interactions and involves classical cadherins (that form adherens junctions), protocadherins, and cadherin-related proteins. Mucin-like protocadherin (also termed cadherin-related family member 5 or mu-protocadherin) has been classified as being cadherin based on sequence homology that is the presence of four cadherin-like repeats; however it also contains the unique extracellular mucin-like domain [3].

This protein has been expressed in the epithelia of the gastroenteric tract, liver, and small and large intestine as well as the kidney [4]. It has been observed mainly at the apical surface of epithelial layers [5, 6]. As expected, the mucin-like protocadherin was found to be involved in epithelial intercellular interactions [7] and in brush border assembly [6]. In early experiments, with L929 fibroblasts transfected with an expression vector that contained* MUPCDH* (the gene for encoding this mucin-like protocadherin), a notable and abnormal aggregation of the transfected cells was observed [7]. This protein's intercellular domain was found to bind *β*-catenin and retain it at the plasma membrane thus resembling the well defined role of E-cadherin in the WNT pathway [5, 8, 9]. *β*-catenin binding by the MUPCDH intercellular domain reduces its transcriptional activity [10].

Decreasing* MUPCDH* gene expression (also known as* CHR5*,* MU-PCDH,* and* MUCDHL*) in colorectal cancer (CRC) has been observed by the Losi et al. [5] study. A loss of protein expression was demonstrated in over 70% tumours [5]. This suggests a tumour suppressor role for protocadherin and is consistent with the observed role of MUPCDH in *β*-catenin binding and WNT signalling. Accordingly,* MUPCDH* overexpression in the CRC HCT116 cell line inhibited* in vitro* proliferationand reduced tumour formation in mice [10].

Downregulation of gene expression in tumours may be related to aberrant epigenetic regulation and an impaired DNA methylation pattern at the 5′ gene promoter region [11]. Over recent years, this phenomenon has drawn much attention in cancer research. Cadherin and protocadherin genes are among those most often sensitive to hypermethylation during neoplastic transformation [8]. Their reduced or silenced expression, resulting from aberrant DNA methylation, occurs frequently in many types of neoplasms, including colorectal tumours [12–15].

This study therefore aims to assess and compare mucin-like protocadherin expression in normal and CRC samples, as well as in tissue sections from the noninvasive adenoma stage. The role of aberrant epigenetic regulation in the downregulation of* MUPCDH* expression was also addressed.

## 2. Materials and Methods

### 2.1. Patients and Tissue Samples

Samples of mRNA were obtained as described previously [16] from the frozen tissues of 26 colorectal adenocarcinomas (AC), 24 corresponding samples of normal colon (NC), and 42 colorectal adenomas (AD) which were used for measuring gene expression by quantitative (q)PCR. Cryostat sections were prepared for each specimen using a Microm HM 505E (Zeiss) and upper and lower sections from each cryosection collection were evaluated by a pathologist for monitoring the relative cell type content.

Formalin-fixed and paraffin-embedded tissue from 24 AC and 12 AD samples were used for DNA isolation and the DNA methylation assessment, whereas 14 of the AC samples with matched normal colonic mucosa sections, along with 10 AD samples, were used for immunohistochemical assessment of mucin-like protocadherin expression.

An additional 10 samples of nonneoplastic colonic epithelia were obtained from normal tissue areas on the margins of resected colorectal tumors acquired from 10 anonymous patients by scraping the colonic epithelial layers with plastic swab sticks. DNA was then isolated followed by DNA methylation analysis which thus constituted the normal controls.

The clinical tissue specimens were collected at the Maria Sklodowska-Curie Memorial Cancer Center and Institute of Oncology in Warsaw in accordance with local ethic committee approval and individual patients' informed consent. Patients' characteristics are presented in Table 1.

### 2.2. Cell Lines Cultures

HCT 116 and HT29 cell lines were cultured in McCoy's 5A medium (Sigma-Aldrich Inc.) supplemented with 10% heat inactivated foetal bovine serum (FBS) (Gibco). SW480 and COLO-205 cell lines were cultured in RPMI-1640 medium (Sigma-Aldrich Inc.) supplemented with 5% and 10% FBS, respectively. All culture media contained 1% antibiotics (Pen Strep, Gibco) and all cell cultures were carried out at 37°C under a humidified 5% CO_2_ atmosphere.

Cells were grown in culture medium until 50% confluence and subsequently treated with 2.5 *μ*M, 5 *μ*M, and 10 *μ*M 5-aza-2′-deoxycytidine (Abcam) followed by 3-day culture. The medium containing 5-aza-dC was refreshed each day. Cells were harvested by scraping.

### 2.3. Quantitative PCR Expression Assessment (qRT-PCR)

Total RNA from tissue samples and cell lines was isolated using the RNeasy Mini (Qiagen). One *μ*g of each RNA sample was subjected to reverse transcription with the Transcriptor First Strand cDNA Synthesis Kit (Roche). qPCR was undertaken using the 384-well format and Applied Biosystems 7900HT Fast Real-Time PCR System (Applied Biosystems) with the Sensimix SYBR kit (Bioline), according to manufacturer's recommendations in a volume of 6 *μ*L.* ACTB* (*β*-actin) was used as reference gene after the evaluation of 4 potential reference genes:* ACTB*,* UBC*,* RPLPO*, and* GAPDH* with the GeNorm software. The standard curves based on amplification of known cDNA template concentrations were used for quantifying the sample PCR products.

### 2.4. Immunohistochemistry (IHC)

Immunohistochemical staining was performed on 4 *μ*m formalin-fixed, paraffin-embedded tissue sections of CRCs and matched normal mucosa from 14 patients, together with 10 colorectal adenomas using the Envision Detection System (DAKO). Sections were deparaffinized with xylene and rehydrated in a series of ethanol solutions decreasing in concentration. Heat-induced epitope retrieval was carried out in a Target Retrieval Solution pH 6 (DAKO) in a 96°C water bath for 20 minutes. After cooling, retrieval solutions were kept at room temperature for 25 minutes and the slides were treated for 5 minutes with an Endogenous Peroxidase Blocker (DAKO). Slides were then incubated with monoclonal antibody against mucin-like protocadherin (PA5-32704, Pierce/Thermo Scientific) at 1 : 300 dilution for 30 minutes at room temperature and subsequently labelled with the Envision Detection System (DAKO). The colour reaction product was developed with 3,3′-diaminobenzidine, tetrahydrochloride (DAB) (DAKO) as substrate and nuclear contrast was achieved with hematoxylin counterstaining. Staining intensity was assessed by a four-grade scale: 0: lack of expression; 1: weak expression; 2: moderate expression; and 3: strong expression. The stained tissue slides were examined by the pathologist blinded to the qRT-PCR results.

### 2.5. DNA Methylation Analysis (Pyrosequencing)

DNA methylation levels of the 5′* MUPCDH* promoter region were analyzed in DNA from 4 cell lines and FFPE derived DNA from 24 cancer samples, 12 adenomas, and 10 normal samples (obtained from swabs of normal colon epithelium). The analysis involved the region located at chr11:624,959-625,053 (grch37/hg19 genome build) that contains 9 particular CpG sites within 95 bp DNA sequence.

The FFPE tissue samples were manually macrodissected to reduce the content of nonneoplastic cells. DNA was isolated using the QIAamp DNA Mini Kit (Qiagen) and bisulfite was converted using EpiTect kit (Qiagen), according to manufacturer's recommendations. The PCR reaction consisted of a 30 *μ*L mixture containing 1x PCR buffer, 2 mM MgCl_2_, 0.25 mM dNTPs, 0.2 *μ*M of each primer, and 0.5 U of FastStart DNA Polymerase (Roche Applied Science) with the following cycling conditions: 94°C for 3 min, followed by 40 cycles of 30 s at 94°C, 30 s at the annealing temperature of 54°C, and 30 s at 72°C with final incubation for 7 min at 72°C. Primers' sequences are shown in Table 2.

The 188 bp long PCR products were analyzed by the PyroMark Q24 System (Qiagen), according to the published protocol [17]. For each sample, the mean methylation level of CpGs located within the analyzed region was used for statistical evaluation.

### 2.6. Chromatin Immunoprecipitation (ChIP)

HCT 116, HT29, COLO205, and HT29 cells were cross-linked for 10 minutes at room temperature by adding formaldehyde directly to the medium (final concentration 1%) followed by formaldehyde quenching upon 5 minutes incubation with 125 mM glycine at room temperature. The fixed cells were suspended in 100 *μ*L of hypotonic buffer A with NP-40 detergent (10 mM HEPES pH 7.9, 2 mM MgCl_2_, 2 mM KCl, NP-40 0.5%) supplemented with protease and phosphatase inhibitors (Thermo; 78441) and incubated on ice for 5 min and then followed by centrifugation, 2000 rpm for 5 min at 4°C. Next, the nuclei pellets were resuspended in lysis buffer (12.5 mM Tris-HCl, pH 8.0; 2.5 mM EDTA; 0.25% SDS) containing protease and phosphatase inhibitors (Thermo; 78441). Chromatin was sheared in a Bioruptor Plus (Diagenode) using a protocol of 30 sec on-off cycles for 10 min at high intensity. ChIP assays were performed using Matrix-ChIP on polypropylene plates as described previously [18]. Immunoprecipitated DNA levels were calculated as percent of input DNA minus the level of DNA immunoprecipitated with nonspecific rabbit IgG or as the ratio of modified histone to total histone H3 [19]. PCR primers for the* MUPCDH* promoter region were used as well as primers for two control promoter regions of transcriptionally active (*ACTB*) and inactive (*HBB*) genes. The following antibodies were used in ChIP assays: nonspecific rabbit IgG (I-1000, Vector Labs), RNA II polymerase (Pol2) (4H8) (Santa Cruz; sc-47701), H3K4me3 (Diagenode; pAb-003-050), H3K27me3 (Millipore, 07-449), and histone H3 (Abcam, ab1791).

### 2.7. Statistical Analysis

Gene expression values and DNA methylation levels were compared by using the two-sided Mann-Whitney *U* test. A significance threshold level *α* = 0.05 was adopted. Data were analyzed and visualized using GraphPad Prism (GraphPad Software).

## 3. Results

### 3.1. The Analysis of* MUPCDH* Expression and DNA Methylation in Patients

**Figure 1:**
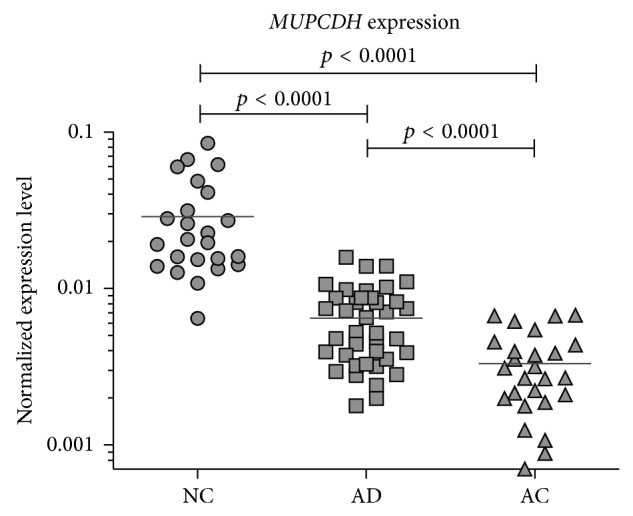
*MUPCDH* expression levels compared between normal colonic mucosa (NC), adenomas (AD), and carcinoma (AC) samples; horizontal lines on graph indicate mean values.

**Figure 2:**
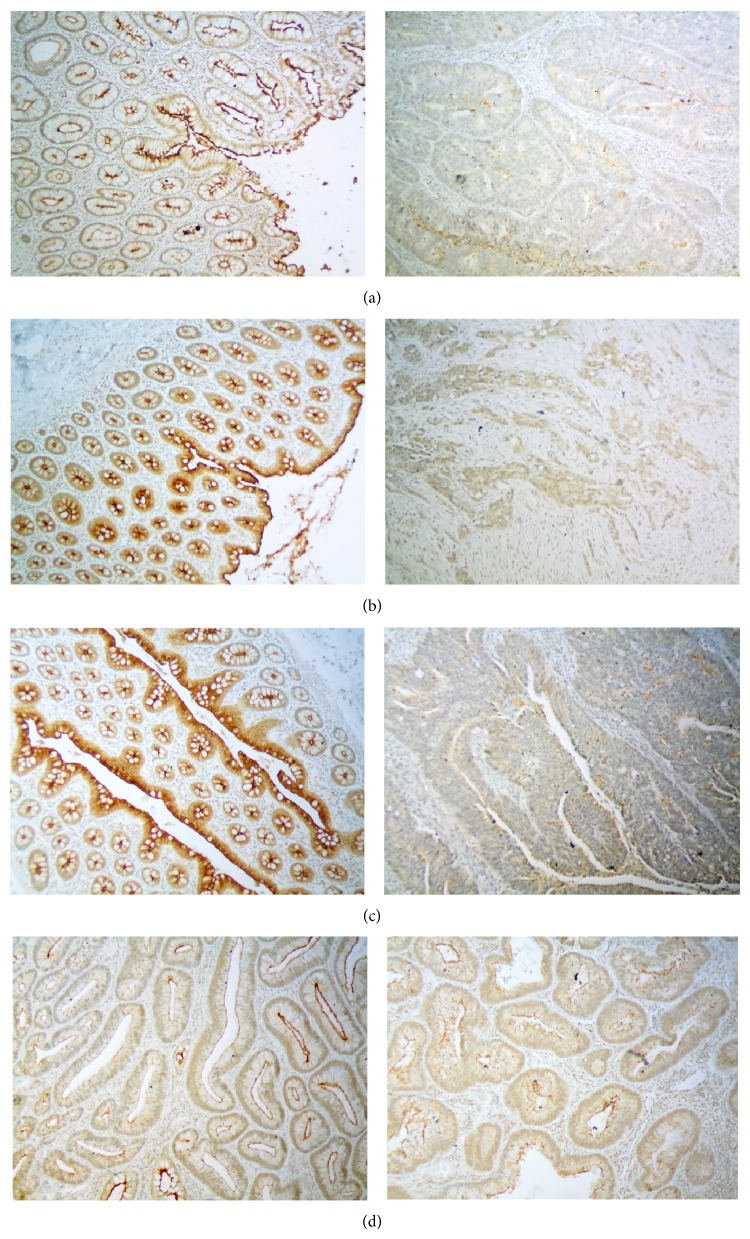
Immunohistochemical staining examples of mucin-like protocadherin in tissue samples. (a–c) Three examples of adenocarcinoma (AC) and matched normal colonic mucosa (NC). Two of the samples were classified as negative for membrane expression (a, b) and one as of low expression (c); (d) two examples of adenomas with moderate mucin-like protocadherin membrane expression; magnification ×100.

Quantitative PCR-based expression analysis demonstrated significant decreases of* MUPCDH* levels in AD samples compared to NC (fold change of expression (FC) = 4.46; *p* < 0.0001) as well as in the AC samples compared to NC (FC = −8.71; *p* < 0.0001). Cancer tissue sections also showed a significant decrease of gene mRNA levels compared to AD (FC = 1.95; *p* < 0.0001) (Figure 1). The results show a stepwise downregulation of gene expression through the subsequent progression stages of colorectal tumours. This trend could be clearly observed in the immunohistochemical staining of AC sections with matched normal samples and adenomas by using the antibody against mucin-like protocadherin and then grading the staining intensity scale. High immunoreactivity was observed primarily for NC sections in the membranes of normal colonic epithelial cells with some degree of polarity in expression. Moderate expression was observed in epithelial cells located at the site of lamina muscularis but was higher in cells located closer to the intestinal lumen. The greatest expression was observed in cells of the apical epithelium layer and those of the crypt lumen. Four AC samples revealed mucin-like protocadherin expression comparable to the matched NC, whereas the expression was clearly lower in the other 10 remaining AC samples. The membrane immunoreactivity was scored as moderate, low, and undetected in 6, 2, and 2 of these AC samples, respectively. Two of the adenoma samples were classified as being of high expression, whereas the other 8 AD samples were assigned as being of moderate expression. Examples are presented in Figure 2.

**Figure 3:**
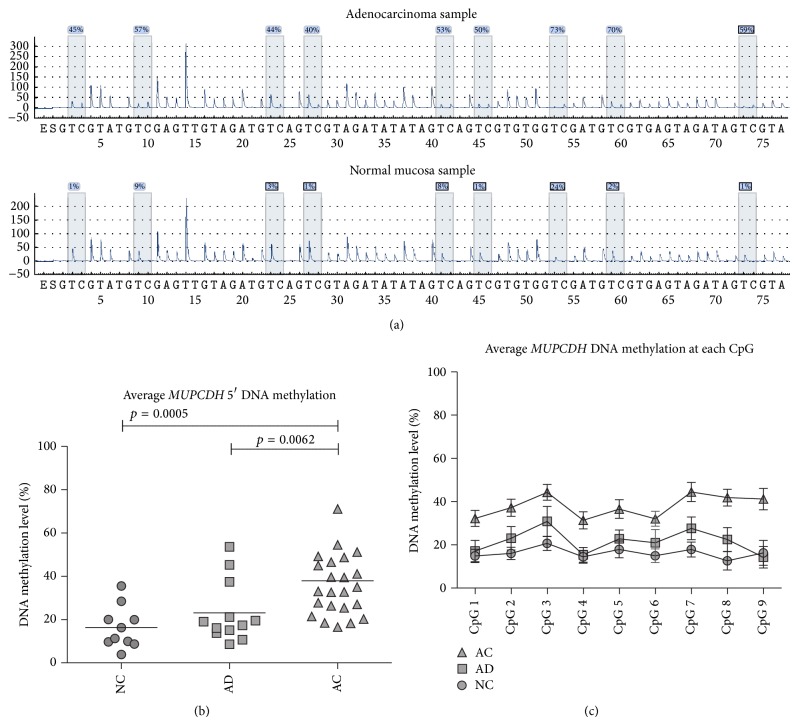
*MUPCDH* promoter DNA methylation results. (a) The examples of pyrosequencing results for individual adenocarcinoma and normal mucosa samples. (b) The average* MUPCDH* promoter DNA methylation in normal colonic mucosa (NC), adenomas (AD), and carcinoma (AC) samples. (c) The average DNA methylation level at each of the analysed CpG positions. Horizontal lines on graph indicate mean values and standard error of the mean.

Carcinogenesis-related changes of gene expression may be mediated by disturbances of epigenetic mechanisms, including aberrant DNA methylation of the 5′ gene promoter region. We focused on DNA methylation at* MUPCDH* 5′ region at the transcription start site that is enriched in CpG dinucleotides. DNA methylation levels were assessed with pyrosequencing in 14 AC, 14 AD, and 10 NC samples. When the three colorectal tissue types were compared, a gradual increase of DNA methylation levels was observed, with the highest* MUPCDH* methylation level in cancer samples. Mean methylation levels were 37.8% (ranging from 16.55% to 88.5%), 23.2% (from 8.6% to 53.6%), and 16.4% (from 3.9% to 35.5%) for AC, AD, and NC, respectively. The* MUPCDH* promoter methylation levels were significantly higher in AC samples than normal mucosa (*p* = 0.0005) and AD (*p* = 0.0062); however the difference between AD and normal samples did not reach significance (Figure 3).

### 3.2. The Role of DNA Methylation in* MUPCDH* Expression in CRC Cell Lines

**Figure 4:**
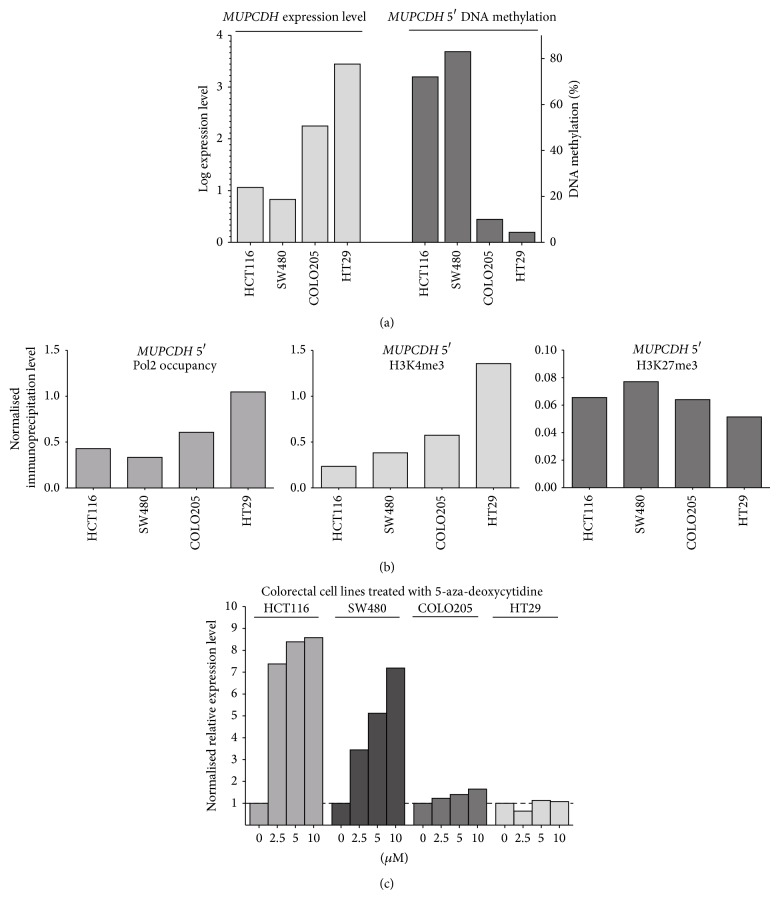
The role of* MUPCDH* 5′ DNA methylation for gene expression in colorectal cancer cell lines. (a) Comparison of* MUPCDH* expression and DNA methylation levels in four cell lines: HCT116, SW480, COLO205, and HT29; (b) results of the chromatin immunoprecipitation assay showing the level of RNA polymerase II binding as well as the levels of H3K4me3 and H3K27me3 at the MUPCDH promoter in four cell lines with diverse gene expression; (c)* MUPCDH* expression levels in colorectal cell lines cultured with various concentrations of DNA methyltransferase inhibitor, 5-aza-2′-deoxycytidine.

In further analysing the relationship between DNA methylation and expression level of* MUPCDH,* we used four CRC cell lines, namely, HCT116, SW480, COLO205, and HT29. High methylation of the gene promoter was observed in HCT116 and SW480, whereas the remaining cell lines were only slightly methylated (Figure 4(a)). Cell lines with high* MUPCDH* DNA methylation displayed low mRNA expression levels in qRT-PCR, whereas COLO205 showed moderate and HT29 revealed high gene expression (Figure 4(a)). Analysing the* MUPCDH* 5′ regions by the ChIP assay demonstrated higher levels of Pol2 in COLO205 and HT29 cells. Furthermore, higher levels of histone H3 lysine 4 trimethylation (H3K4me3, being a marker of transcriptionally active chromatin) were also observed in these two cell lines when compared with HCT116 and SW480. The levels of the repressive histone marker, the H3K27me3 at* MUPCDH* promoter, were the lowest in HT29 cells, which was in keeping with the highest* MUPCDH* mRNA expression for this cell line (Figure 4(b)).

All four cell lines were cultured with increasing concentrations of DNA methyltransferase inhibitor 5-aza-2′-deoxycytidine (i.e., 2.5 *μ*M, 5 *μ*M, and 10 *μ*M). An increased* MUPCDH* expression in HCT116 and SW480 was observed in cultures with 5-aza-dC compared to cells cultured without inhibitor. The highest increase of expression was observed for the 10 *μ*M 5-aza-dC medium concentration. HCT116 and SW480 showed 8.2- and 7.3-fold change in expression, respectively, compared to the corresponding cells cultured without 5-aza-dC (Figure 4(c)). The increase of* MUPCDH* expression was not observed in COLO205 and HT29 cells after 5-aza-dC treatment (Figure 4(c)).

## 4. Discussion

Mucin-like protocadherin is a tissue specific cell adhesion protein that is expressed mainly in the epithelium of the gastrointestinal tract [20]. This protein is involved in intercellular interactions either through the extracellular cadherin domain or by intercellular signaling via *β*-catenin binding to the plasma domain [10].

Using IHC we observed that mucin-like protocadherin is expressed at high levels in normal colonic epithelium and the expression pattern has a certain asymmetry in an epithelium cross section. The level of this protein is relatively low at the lateral border but highest at the apical surface of the epithelial layer in the enterocytes that form the brush border; this observation is being consistent with those previously reported [5, 6, 10].

By comparing NC with AD and AC sections, the presented study has found a gradual decrease of* MUPCDH* expression that progresses during the so-called “adenoma-carcinoma sequence.” However, this was not observed in a previous study on the role of mucin-like protocadherin in carcinoma pathogenesis where only cancer samples were compared with normal colonic mucosa sections [5]. The finding that mucin-like protocadherin is downregulated at an early stage of colorectal tumourigenesis agrees with its previously reported suppressive role in WNT signalling through *β*-catenin retainment. A disturbed WNT/*β*-catenin pathway is considered as being the initiating event in colorectal tumourigenesis with the well described role of early occurring* APC* mutations in sporadic colorectal tumours [21]. Previous experimental studies have shown that the intercellular mucin-like protocadherin domain binds *β*-catenin and prevents its transposition to the cell's nucleus and transcription factor activity [9, 10]. In this respect, cadherin may be considered as a WNT inhibitor and its downregulation in the early stages of colorectal tumours is consistent with the reported dominant role of the WNT/*β*-catenin pathway in the initiation of colorectal neoplastic transformation.

The possibility that aberrant epigenetic regulation plays a role in the downregulation of* MUPCDH* expression in CRC has been discussed recently in a review by Losi and Grande [20]. However, to date, no experimental data has been reported.

The current study has assessed the DNA methylation profile of the* MUPCDH* 5′ region in normal, adenoma, and cancer sections of the colon with pyrosequencing of bisulphite-converted DNA that allows for quantitative assessment of methylation levels in the samples. Generally, low DNA methylation levels were observed in normal samples but were increased in adenomas and more prominently in cancer samples. This increase of DNA methylation in tumour samples corresponds to decreasing levels of gene expression, thus suggesting that elevated DNA methylation plays a role in* MUPCDH* downregulation and follows the generally accepted model of epigenetic inactivation of tumour suppressor genes in carcinogenesis [11].

High promoter DNA methylation was observed in two of the four analyzed colorectal cancer cell lines: HCT116 and SW480. These cells demonstrated low* MUPCDH* mRNA expression levels and lower Pol2 occupancy at the* MUPCDH* promoter in comparison to COLO205 and HT29, which in turn showed low promoter DNA methylation and higher Pol2 density. The corresponding profile of an active chromatin marker that is H3K4me3 was observed in the analyzed cell lines with the highest H3K4me3 levels in HT29 cells which was reflected by the highest* MUPCDH* mRNA in the CRC cell lines tested. HT29 also showed the lowest level of H3K27me3, a marker of inactive chromatin. Therefore, when comparing these four cell lines,* MUPCDH* expression levels are shown to be related to the particular DNA methylation level and chromatin status at the gene promoter region that could be so expected based on the generally accepted model of gene transcription regulation [11]. This result is also consistent with the analysis of normal and tumour tissue samples, where high DNA methylation in cancer is related to low* MUPCDH* levels.

In a further investigation of the role of DNA methylation in the regulation of* MUPCDH* expression, the four previously mentioned cell lines were treated with 5-aza-dC. This is a widely used inhibitor of DNA methyltransferases for restoring the expression of epigenetically silenced genes [22]. The increase of* MUPCDH* expression was found in those cell lines with high promoter methylation, whereas it was very slight or not visible in cells exhibiting low DNA methylation levels. This result thus supports the observation that the gene expression depends on DNA methylation.

Our findings show that it is not clear if the increased DNA methylation of the studied promoter region is the cause or consequence of* MUPCDH* gene silencing. Using the DNA methyltransferase inhibitor gave increased gene expression in the cell lines with a highly methylated promoter. However when comparing the NC, AD, and AC samples, a notable decrease of gene expression was found in AD compared to normal mucosa, whereas most ADs showed similar levels of promoter methylation to normal samples. Some cancer samples also demonstrated moderate DNA methylation levels that are similar to the normal samples, whereas gene expression levels were significantly lower in AC. This may suggest that the increased DNA methylation of the analyzed region is not the only factor that downregulates* MUPCDH* expression or that it is a secondary event that stabilizes this gene silencing process and contributes to the closing of chromatin at the* MUPCDH* locus.* MUPCDH* expression has been previously found to be positively regulated by the Cdx2 homeobox. Cdx2 acts as a suppressor in animal models of CRC and its expression may be decreased during tumourigenesis [23]; however its role in human cancer is still unclear [24].

## 5. Conclusion

The results indicate that downregulation of mucin-like protocadherin expression occurs in early stages of colorectal tumourigenesis and progresses during the adenoma-carcinoma sequence. Epigenetic mechanisms, including aberrant DNA methylation of* MUPCDH* 5′ region, are involved in the silencing of this gene expression.

## Figures and Tables

**Table 1:** Patients' characteristics.

	Expression qRTPCR	Expression IHC	DNA methylation pyrosequencing
*Cancer patients *			
Number of patients	26	14	24
Sex (number of patients)			
Male	11	6	10
Female	15	8	14
Age (years)			
Range	38–82	38–78	38–82
Median	64	67	63.5
Astler-Coller's stage (number of patients)			
B1	4	3	4
B2	15	8	13
C2	7	3	7
*Adenoma patients *			
Number of patients	42	12	12
Sex (number of patients)			
Male	25	6	6
Female	17	6	6
Age (years)			
Range	41–83	42–80	42–80
Median	60	61.5	61.5
Size (mm)			
Range	9–50	10–50	10–50
Median	15	16	16
Histopathology (number of patients)			
Tubular AD	29	5	5
Tubulovillous AD	13	7	7
*Normal mucosa *			
Number of samples	24	14	10

**Table 2:** PCR primers' sequences.

Gene symbol	Primer	Sequence 5′ → 3′
qRT-PCR		
*MUPCDH *	Forward	CTTCTACGCAGAGGTTGAGG
	Reverse	GGGCTCCTGTTCGGAAAC
*ACTB *	Forward	AGAGCTACGAGCTGCCTGAC
	Reverse	AAGGTAGTTTCGTGGATGCC

Pyrosequencing		
*MUPCDH *	Forward	TTGGTAGTGGGTTGGATTAG
	Reverse	Biot-AAACCCAAAACCCCATCTTA
	Sequencing	GTGGGTTGGATTAGT

Chip assay		
*MUPCDH *	Forward	CGCTCT CCAGTC CCTTCT G
	Reverse	GACTGA GTGCAG GGGTCA AC
*ACTB *	Forward	ACGCCTCCGACCAGTGTT
	Reverse	GCCCAGATTGGGGACAAA
*HBB *	Forward	GCAATAGATGGCTCTGCCCT
	Reverse	GACAGGTACGGCTGTCATCA
